# Draft Genome Sequence of the Fish Pathogen *Mycobacterium pseudoshottsii* Strain JCM15466, a Species Closely Related to *M. marinum*

**DOI:** 10.1128/genomeA.01630-15

**Published:** 2016-02-11

**Authors:** Jun-ichi Hikima, Masahiro Sakai, Takashi Aoki, Haruko Takeyama, John Hawke, Kazuki Mori, Kosuke Tashiro, Satoru Kuhara

**Affiliations:** a Department of Biochemistry and Applied Biosciences, Faculty of Agriculture, University of Miyazaki, Miyazaki, Japan; b Integrated Institute for Regulatory Science, Research Organization for Nao and Life Innovation, Waseda University, Tokyo, Japan; c Department of Life Science and Medical Bioscience, Waseda University, Tokyo, Japan; d Department of Pathobiological Sciences, School of Veterinary Medicine, Louisiana State University, Baton Rouge, Louisiana, USA; e Department of Bioscience and Biotechnology, Faculty of Agriculture, Kyushu University, Fukuoka, Japan

## Abstract

*Mycobacterium pseudoshottsii* is a slowly growing photochromogenic mycobacterium and fish pathogen isolated from wild marine fishes. *M. pseudoshottsii* closely resembles *M. marinum*, which is a human and animal pathogen. Here, we report the draft genome sequence of *M. pseudoshottsii* strain JCM15466, originally isolated from striped bass, *Morone saxatilis*.

## GENOME ANNOUNCEMENT

Mycobacteria are members of the family *Mycobacteriaceae* in the order *Actinomycetales*. They are pleomorphic, Gram-positive, acid-fast, nonmotile, nonsporulating, and rod shaped (1). The genus *Mycobacterium* currently contains 148 recognized species, including *M. marinum*, *M. pseudoshottsii*, and an unidentified *Mycobacterium* sp., which are the most commonly reported bacterial fish pathogens (2). Some are also zoonotic, causing cellulitis, skin tuberculosis, and foreign body reaction in humans (3).

In 2005, *M. pseudoshottsii* was initially isolated from striped bass (*Morone saxatilis*) from Chesapeake Bay (4). However, both the range of host species and area of disease distribution have expanded to a variety of fishes and locations (5). Of the several *Mycobacterium* species isolated from fish, it has been suggested that *M. marinum*, *M. shottsii*, *M. pseudoshottsii*, and *Mycobacterium* spp. are closely related species based on molecular phylogenetic analysis using 16S rRNA, hsp65, and rpoB genes and ITS region (4, 6–9). However, all of these genes are almost completely identified within the above-mentioned *Mycobacterium* spp., with a few substitutions (7, 8). Therefore, to clarify the phylogenetic relationship between *M. pseudoshottsii* and other species, the whole genome of *M. pseudoshottsii* strain JCM15466 was determined and analyzed in this study. *M. pseudoshottsii* strain JCM15466^T^ causes the same disease as reported in striped bass from Chesapeake Bay. The strain JCM15466 (RIKEN Japan type strain) is also named L15 (original strain) (4), ATCC BAA-883 (USA), NCTC 13318 (UK), and DSM 45108 (Germany).

Genomic DNA of the fish-pathogenic strain *M. pseudoshottsii* JCM15466 was extracted by the method described previously (10). The draft genome of *M. pseudoshottsii* JCM15466 was obtained using MiSeq 500 v2 (paired-end, 250-bp reads) (Illumina, Inc., San Diego, CA, USA). A total of 2,127,506 high-quality paired-end reads were obtained, and 916,416 Mb reads were assembled by Platanus genome assembler version 1.21 (http://platanus.bio.titech.ac.jp/platanus/; Tokyo Institute of Technology, Tokyo, Japan) (11). Coding sequences (CDSs) of the draft whole-genome sequence of *M. pseudoshottsii* JCM15466 were predicted and annotated using Glimmer version 3.02 (http://www.ncbi.nlm.nih.gov/genomes/MICROBES/glimmer_3.cgi) and the RAST server (12). The genome sequence of *M. marinum* strain M (GenBank accession number NC_010612), isolated from human, was used as a reference for these analyses. A total of 223 assembled contigs (i.e., >1 kb) were obtained, and the total length was approximately 5.92 Mb, including an approximately 0.18-Mb unmatched sequence to the *M. marinum* strain M genome (the estimated total length was approx. 5.74 Mb).

The genome of *M. pseudoshottsii* JCM15466 contains 5,710 CDSs classified into 27 subsystems, of which 3,646 CDSs were classified as unknown genes of hypothetical function. Among 5,710 CDSs, 483 genes were categorized as amino acids and derivates; 417 genes as fatty acids, lipids, and isoprenoids; 392 genes as cofactors, vitamins, prosthetic groups, and pigments; and 461 genes associated with carbohydrates. Furthermore, a total of 168 genes were found to be associated with virulence, disease, and defense. In the genome of *M. pseudoshottsii* JCM15466, numerous mutations and substitutions were found in proline-glutamate or proline-proline-glutamate genes, and it contains genes related to virulence, disease, and defense subsystems as reported in *Mycobacterium* sp. 012931 (13).

### Nucleotide sequence accession numbers.

The whole-genome sequences of *M. pseudoshottsii* JCM15466 (L15) were deposited in DDBJ under the accession numbers BCND01000001 to BCND01000247 (total of 247 entries).
